# Synthesis, Characterization, Theoretical and Experimental Anticancer Evaluation of Novel Cocrystals of 5-Fluorouracil and Schiff Bases against SW480 Colorectal Carcinoma

**DOI:** 10.3390/pharmaceutics15071929

**Published:** 2023-07-11

**Authors:** Farhat Jubeen, Ishrat Jabeen, Usman Aftab, Sadia Noor, Mah e Hareem, Misbah Sultan, Mohsin Kazi

**Affiliations:** 1 Department of Chemistry, Government College Women University, Arfa Kareem Road, Faisalabad 38000, Pakistan; dr.farhatjubeen@gcwuf.edu.pk; 2 School of Interdisciplinary Engineering and Sciences (SINES), National University of Sciences and Technology (NUST), Sector H12, Islamabad 44000, Pakistan; ishrat.jabeen@sines.nust.edu.pk (I.J.); sadiaa613@gmail.com (S.N.); mah.e.hareemkayani@gmail.com (M.e.H.); 3 Department of Pharmacology, University of Health Sciences, Lahore 54600, Pakistan; usmanaftab@uhs.edu.pk; 4 Department of Inorganic Chemistry, University of Hohenheim, 70599 Stuttgart, Germany; 5 Institute of Chemistry, University of the Punjab, Quaid-i-Azam Campus, Lahore 54590, Pakistan; 6 Department of Pharmaceutics, College of Pharmacy, King Saud University, P.O. Box 2457, Riyadh 11451, Saudi Arabia

**Keywords:** Schiff bases, MTT assay, computational study, 5-FU, cocrystallization

## Abstract

The chemotherapeutic agent known as 5-fluorouracil (5-FU) is an artificial fluoropyrimidine antimetabolite that has been widely used for its antineoplastic properties. Cocrystals of 5-fluorouracil (5-FU) with five different Schiff bases (benzylidene-urea (**BU**), benzylidene-aniline (**BA**), salicylidene-aniline (**SA**), salicylidene-phenylhydrazine (**SPH**), and para-hydroxy benzylideneaniline (**HBA**)) are reported in this study. The newly synthesized cocrystals were analyzed by FTIR and PXRD. In this study, we investigated the antitumor efficacy of 5-FU derivatives in SW480 colon cancer cells via MTT assay at varying dose concentrations. Molecular docking was performed to predict the binding mechanism of TS with various 5-FU complexes. FTIR revealed the presence of respective functional groups in the prepared cocrystals. The frequencies (v) of N-H (3220.24 cm^−1^) and carbonyl groups (1662.38 cm^−1^) in the spectrum of 5-FU shifted considerably in all derivative cocrystal new interactions. There was a noticeable transformation in the PXRD peak of 5-FU at 2θ = 28.37° in all derivatives. The novelty of the present study lies in the fact that 5-FU-BA showed an anticancer potential IC_50_ (6.4731) far higher than that of 5-FU (12.116), almost comparable to that of the reference drug doxorubicin (3.3159), against SW480 cancel cell lines, followed by 5-Fu-HBA (10.2174). The inhibition rates of 5-FU-BA and 5-FU-HBA were highest among the derivatives (99.85% and 99.37%, respectively) in comparison with doxorubicin (97.103%). The results revealed that the synthesized 5-FU cocrystals have promising antitumor efficacy compared with previously reported 5-FU and 5-FU. The activities of the cocrystals were rationalized by a molecular modeling approach to envisage binding modes with the target cancer protein.

## 1. Introduction

5-fluorouracil (5-FU), an artificial fluoropyrimidine antimetabolite chemotherapeutic agent, one of the forerunners and frequently practiced drugs showing key antineoplastic potential, was first reported in 1957 by Heidelberger et al. [1]. It is effective in the management and systemic chemotherapy of solid tumors such as colorectal, stomach, lung, ovarian, and breast cancers. However, the hampered use of this potential antineoplastic drug because of poor pharmacokinetics has framed it in the extensively studied following top four suggested and top selling anticancer drugs: revlimid, rituximab, trastuzumab, and bevacizumab [2]. Despite the intrinsic pitfalls of low absorptivity and high solubility leading to the short lifespan of 5-FU, its administration is a real challenge. Thanks to its nondirectional mode of action and fatal toxic effects on noncancerous body cells, its toxicity profile includes stomatitis, nausea, puking, alopecia, diarrhea, neurologic defects, and even bone marrow and cardiotoxicity [3,4,5]. Strategies for the prevention or treatment of 5-FU-related toxicities could be made possible through effective general and specific supportive approaches [6]. Over the past few decades, several modulation approaches using 5-FU-based combination regimens and 5-FU pro-drugs have been used to elevate anticancer activity to overcome clinical resistance [7,8].

Using a molecular hybridization approach, the pharmacophores of genistein and 5-FU were studied as new therapeutic agents with anticancer potential against colorectal carcinoma [9]. To improve the biological half-life and ratio of apoptosis of the drug, nanocarriers of chitosan with 5-FU were employed [10]. To improve the physicochemical characteristics, three synthesized drug components, functionalized folinic acid, coumarin derivative, and 5-FU, were found to follow pseudo-first-order kinetics for controlled drug release [11]. To combat colorectal cancer, 5-FU-loaded magnetic nanoparticles were synthesized by Yusefi [12].

Schiff bases are imine or azomethine (-C=N-) functionalities bearing predominantly synthetic products of the condensation reaction between primary amines and active aromatic carbonyl moieties employing suitable solvents, first reported by Hugo Schiff [13]. Legion applications of these bases have been reported as sequestering or chelating agents [14,15], catalysts [16,17], polymerization initiators [18], and fluorescent materials [19]. These compounds are used as drug agents, biological probes, or analytical tools for biological or therapeutic applications [20]. Schiff bases also exhibit biological activities ranging from antibacterial [21,22] to antifungal [14,23], anti-inflammatory [24], antimalarial [25], antiviral [26], antiproliferative [27], and antipyretic properties [28]. A Schiff base of 5-FU as a Ketone and o-amino aniline complexed with various metals was studied for antimicrobial and antioxidant potential [29] but not for anticancer potential. The emergence of the anticancer potential of Schiff bases and their corresponding complexes has fascinated researchers and led them to launch new anticancer drugs with minimal or no side effects. This breakthrough characteristic of the anticancer activity of Schiff bases is more targeted, while most anticancer drugs exhibit toxicity because of a lack of cell target specificity. Schiff bases work synergistically with other anticancer drugs to show improved anticancer activity and minimal side effects. There are many reports to support the remarkable synergistic therapeutic efficacy via controlled drug delivery employing Schiff bases, such as combined treatment of metformin and 5-FU coloaded hydrogels in the treatment of colorectal cancer [30]. Assembly via Schiff base bonding of pH-responsive dopamine nanoparticles with high synergy in anticancer efficacy has also been reported [31]. It has been implicated that the nitrogen atom of azomethine can develop hydrogen bonding with the functional sites of cellular components and interfere with normal cell processes [32]. The same feature of Schiff bases makes them an effective scaffold or pharmacophore to design cocrystals through van der Waal interactions to supplement the potency of existing anticancer drugs.

Contemporarily, 5-FU has significant potential for cocrystallization of pharmaceutically active components, as it has both hydrogen donors and hydrogen acceptors within the structure, resulting in enhanced solubility, dissolution, permeability, stabilization, and bioavailability of unstable molecules through intermolecular interactions [33,34]. Cocrystallization of 5-FU with improved cytotoxic activity with physiologically active urea, thiourea, acetanilide, and urea has been reported by Jubeen et al. [35]. To the best of our knowledge, this is the first study reporting successful cocrystallization of 5-FU with five novel Schiff bases to improve the therapeutic potential of 5-FU. In the present study, we have successfully prepared some novel cocrystal prodrugs of 5-FU using selectively synthesized Schiff bases as coformers (benzylidene-aniline, benzylidene-urea, salicylidene-aniline, salicylidene-phenylhydrazine, and para-hydroxy benzylideneaniline), which have not yet been reported to the best of our knowledge. The advantages of cocrystallization of 5-FU with Schiff bases have been explored to examine their in vitro anticancer potential. Therefore, cocrystals were prepared to augment the anticancer potential by ameliorating the physicochemical performance of 5-FU to achieve persistent drug release and long-term efficacy. Using the same cocrystallization strategy, a unique zwitterionic cocrystal of 5-FU and L-proline (PL) was successfully prepared, followed by its incorporation into PEG-PCL carriers to obtain cocrystal micelles [36]. Four new cocrystals of 5-FU were prepared with isomeric hydroxybenzoic acids, including salicylic acid, 3-hydroxybenzoic acid, and 4-hydroxybenzoic acid, and studied for their membrane permeation ability. Cocrystallization enhanced their lipid solubility, which in turn improved their membrane permeability of the transdermal drugs [37]. Our group has previously reported cocrystals of 5-FU using four different cyclic dimers of amino acids (alanine, glycine, leucine, and tryptophan) to improve their anticancer efficacy [38] and, in a discrete study, successful cocrystallization of 5-FU with organic acids, namely, succinic acid, cinnamic caid, malic acid, and benzoic acid, is reported [39]. All of the cocrystals demonstrated anti-HCCT-116 activity when examined via anticancer MTT assays against HCCT-116 cell lines. Thanks to the innate feature of 5-FU to disturb the base pairing for H-bonding in DNA to explore and synergistically improve antineoplastic potential [40], cocrystals of 5-FU with aza donors such as acridine, phenazine, and bispyridylethene are reported by Delori [41]; 1:1 cocrystals of 5-FU with ethylhypoxanthine by Kim [42]; and cocrystallization of 5-FU with biologically active amino acids by Wang [43]. The novelty of the present study lies in the fact that all of the synthesized Schiff bases are reported for the first time to develop prodrugs of f-Fu and, among them, 5-Fu-benzylidene-aniline and 5-Fu-para-hydroxy benzylideneaniline showed anticancer potential far higher than that of native 5-Fu, almost comparable to that of the reference drug doxorubicin. One significant aspect of this study is the interpretation of molecular interactions using Molecular Operating Environment (MOE) software. The computational results revealed that, compared with pure 5-FU, eminent solid formulations were shown by the cocrystals, indicating their promising anticancer effectiveness. All of the cocrystals showed interaction via H-bonding and the theoretical calculated interactional energies are helpful in revealing the anticancer potential of the cocrystals.

## 2. Materials and Methods

### 2.1. Chemicals

5-FU (Alfa-Aesar, Ward Hill, MA, USA, purity 99%), distilled water, ethanol (Merck KGaA, Darmstadt, Germany, purity 99.5%), glacial acetic acid (Dejung, South Korea, purity 98%), phenyl hydrazine (Sigma Aldrich, St. Louis, MO, USA, purity 97%), benzaldehyde (Sigma-Aldrich purity 99.5%), para-hydroxy benzaldehyde (Alfa-Aesar, Gongduk-Dong, South Korea, purity 98%), aniline (Sigma-Aldrich, St. Louis, MO, USA, purity 99.5%), salicylaldehyde (Alfa-Aesar, Gongduk-Dong, South Korea, purity 99%), and urea (Alfa-Aesar, Gongduk-Dong, South Korea, purity 93%) were obtained from local chemical vendors. All compounds were used as received without any further purification.

### 2.2. Synthesis of Schiff Bases

**Scheme 1:**
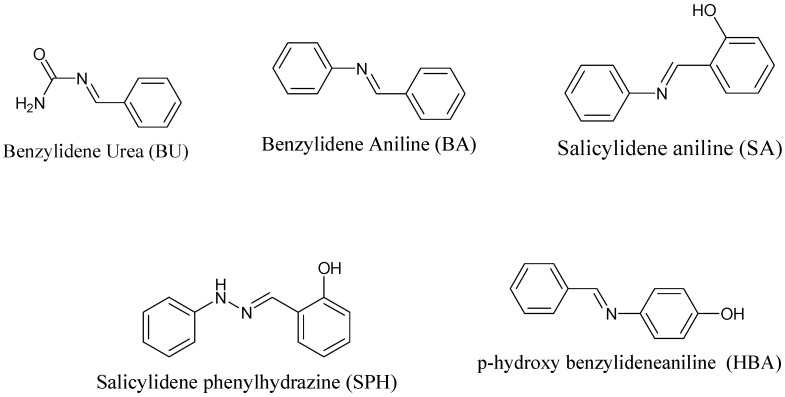
Structures of Schiff bases synthesized using urea, aniline, and phenyl hydrazine as amines and benzaldehyde, ortho-hydroxy benzaldehyde, and p-hydroxy benzaldehyde as aldehydes.

The synthesis of the desired Schiff bases was carried out following the thermal condensation method. All of the Schiff bases, including benzylidene-urea (**BU**), benzylidene-aniline (**BA**), salicylidene-aniline (**SA**), salicylidene-phenylhydrazine (**SPH**), and para-hydroxy benzylideneaniline (**HBA**), were synthesized using equimolar ratios of amines (0.1 M) (aniline 9.1 mL, phenyl hydrazine 9.8 mL, and urea 6 g) and aldehydes (benzaldehyde 10.2 mL and salicylaldehyde 12.5 mL). The structures of the Schiff bases are shown in Scheme 1. The reactants were mixed and stirred at 100 °C for 30 min in a round bottom flask and solvated by absolute ethanol (13–15 mL) in the presence of 1–2 drops of glacial acetic acid. Pure crystals of Schiff bases were obtained from ice-cold solutions and the products were recrystallized from 50% ethanol [44].

### 2.3. Synthesis of 5-FU-Containing Cocrystals

After the successful formation of Schiff base precursors, 5-fluorouracil cocrystals were obtained by adopting the grinding method. Appraised equimolar amounts of 5-FU (4.4 mM; 0.572 g) and co-former Schiff bases, benzylidene-urea 0.65 g (**BU**), benzylidene-aniline 0.79 g (**BA**), salicylidene-aniline 0.86 g (**SA**), salicylidene-phenylhydrazine 0.93 g (**SPH**), and para-hydroxy benzylideneaniline 0.86 g (**HBA**), were stirred vigorously for 30 min. The fine powders were then dissolved in distilled water followed by slow evaporation of water to yield the pure crystalline product.

### 2.4. FTIR and PXRD Analyses

The synthesized cocrystals were analyzed using FTIR (Shimadzu IR prestige-21, Columbia, MD, USA) and PXRD (D2 PHASER—Bruker, Billerica, MA, USA) to study the formation of precursor Schiff bases as well as H-bonding between the active pharmaceutical ingredient (API) and co-formers. Fourier transform infrared (FTIR) (Agilent Technologies Corp., 630, Santa Clara, CA, USA) analysis was employed to detect variation in the vibrational modes of all functional groups responsible for Schiff base formation followed by cocrystal formation. The formation of cocrystals of all precursor Schiff bases with 5-FU was confirmed through comparative spectral analysis with pure 5-FU, and the relocation of amine and carbonyl functionalities from normal vibrational expansions verified the noncovalent cocrystallization connections. PXRD D2 PHASER-Bruker was used to study the crystallinity of the Schiff bases and analyses of the cocrystals. This analysis helps in determining the constructive interaction of monochromatic X-rays and crystal particles. The X-rays were generated by cathode ray tubes followed by their purification to obtain monochromatic photons.

### 2.5. In Vitro MTT Antitumor Bioassay

The synthesized cocrystals were evaluated for their antitumor potential using the ATCC^®^CCL-247TM SW-480 colon cancer cell line following the MTT assay [45,46]. All sample cells were grown in 96-well culture plates in Dulbecco’s Modified Eagle Medium (DMEM). The samples were supplied with 10% fetal bovine serum and 1% of each of streptomycin and penicillin-G to the medium and incubated under humid conditions at 37 °C for 24 h and 5% CO_2._ Once a confluent monolayer was formed, trypsinization of the actively dividing cells was carried out. When a cell suspension with a cell count of 10^5^ cells/mL was attained, it was seeded in the culture media using different concentrations (200 µg/mL, 100 µg/mL, 50 µg/mL, 25 µg/mL, 12.5 µg/mL, and 6.25 µg/mL) of 5-FU and its corresponding cocrystals (samples 1–6). The sample plate was incubated for 48 h under a 5% CO_2_ environment at 37 °C. Afterwards, the cell viability was measured at all concentrations of samples 1–6. Subsequently, 20 µL of 3-(4,5-dimethylthiazol-2-yl)-2,5-diphenyl-2*H*-tetrazolium bromide (MTT) dissolved in 5 mg/mL PBS was transferred to each well, followed by incubation of the plate under the aforementioned conditions.

The cell monolayers were carefully separated after incubation and loaded with 100 µL of dimethyl sulfoxide (DMSO) to solubilize formazan crystals formed because of metabolically active cells. The optical density (O. D) of the wells was measured at 570 nm using a microplate reader, with 655 nm as the reference. Doxorubicin was the reference standard/positive control in the assay. The numerical magnitude of IC_50_ was calculated from the dose-dependent curve. The following formula was employed to estimate the growth inhibition rate at each dilution:

$$\mathrm{Inhibition}\;\mathrm{rate}=\frac{O.D(control\;sample\;well)-O.D(treated\;sample\;well)}{O.D(control\;sample\;well)}\times 100\%$$

### 2.6. Protein Structure Selection and Preparation

Structure selection is the crucial step in molecular modeling. Therefore, the X-ray crystallographic structure of thymidylate synthase (TS) was taken from the Protein Data Bank (PDB) with ID 1HVY and a resolution of 1.90 Ǻ [47]. The structure was imported into the Molecular Operating Environment (MOE) for energy minimization. The structure was cleaned and energy was minimized using AMBER 99 forcefield to be further used as a receptor for molecular docking analysis.

### 2.7. Ligand Data Collection

The 3D structures of benzylideneaniline, benzylideneurea, salicylideneaniline, salicylidenephenyl-hydrazine, and hydroxybenzylideneaniline in complex with 5-FU were built using ChemDraw Ultra 8.0.

### 2.8. Molecular Docking

Molecular docking was performed to predict the binding mechanism of TS with various 5-FU complexes. Energy-minimized TS structures along with ligands were imported into Genetic Optimization for Ligand Docking (GOLD suite v 5.3) [48] to perform molecular docking. GOLD calculates the fitness score, which is a sum of various energy terms, including internal and external hydrogen bond energies (HB_int, HB_ext), internal and external van der Waals energies (VDW_int, VDW_ext), and internal torsion (TOR_int), as given in the formula below:

GOLD_Score = ∆G (HB_int) + ∆G (HB_ext) + ∆G(VDW_int) + ∆G (VDW_ext) + ∆G (TOR_int)

The binding site of TS was identified through the literature, while the cocrystallized structure of TS was also visualized in MOE to identify the important binding site residues. However, binding pocket residues were selected from previous in silico interaction studies of TS with 2′-deoxyuridine 5′-monophosphate (dUMP), 5-FU, and quinozoline antifolate derivatives [35,49,50]. Docking calculations were carried out by setting the coordinates as X = 5.0782, Y = 11.4077, and Z = 18.8835, with an area of 15 Å covering all of the specific binding site residues, including Arg50, Phe80, Leu108, Asn112, Leu192, Cys195, His196, Gln214, Arg215, Ser216, Asp218, Gly220, Leu221, Gly222, Phe225, Asn226, His256, and Tyr258, as their significance has been reported in the literature. The first docking was performed with 5-FU and 100 poses per ligand were generated. The best docked pose in terms of the highest gold fitness score and interaction was selected, the second input of docking was given with each of the five ligands, and 10 poses per ligand were generated. The docking results were then analyzed and the interaction analysis was performed.

### 2.9. Computational Studies

The activities of the cocrystals were rationalized by a molecular modeling approach to envisage binding modes with the target cancer protein. The calculated interaction energies were later used to reveal the anticancer potential of the cocrystals.

## 3. Results and Discussion

### 3.1. Fourier Transform Infrared (FTIR) Analysis

Vibrational modes of functional groups were examined in the infrared region to analyze the chemical bonds. The precursor Schiff bases and cocrystals of 5-FU with these precursors were analyzed by FTIR to observe the shifts in energetic vibrations in the H-bond forming potential functional groups. FTIR spectral analysis facilitated the assignment of characteristic absorption peaks to identify the corresponding compound. Initially, the variations in the vibrational interaction in functional groups in the precursor Schiff bases were followed by their H-bonding interactions. All the spectra were drawn in the range of 4000–400 cm^−1^ per KBr pellet.

### 3.2. FTIR Analysis of Schiff Bases

The principal functional group in all Schiff bases is the imino group located in the 1680–1580 cm^−1^ range (-C=N-). The characteristic peak frequency of -C=N- with physical appearance of all the synthesized precursor Schiff bases are evinced in Table 1. Two aromatic rings of aniline and benzylidene in the benzylideneaniline (BA) molecule are linked by an azomethine (-C=N-) functionality [51]. The formation of the benzaldehyde-aniline Schiff base was confirmed by the presence of a band at 1606 cm^−1^ owing to the -C=N- bond, and its corresponding stretching peaks were observed at 1090 to 1020 cm^−1^. The aromatic ring peaks were present at 1530 cm^−1^ and 1508.8 cm^−1^. In the benzaldehyde-urea Schiff base (BU), the >C=N- functionality was present at 1613 cm^−1^, whereas its corresponding stretching peak was observed at 1076 cm^−1^. In addition, there was also a strong intensity peak for the C=O bond at 1662 cm^−1^ (Figure S1, Supplementary File).

In the case of the salicylidene-aniline (**SA**) Schiff base, the presence of a peak at 1592.61 cm^−1^ confirmed imine functionality, whereas bending vibrations were observed at 1625–1609 cm^−1^. The peaks indicating C-O and C-N functional groups were observed at 1258 cm^−1^ and 1125 cm^−1^, respectively, while a broad peak at 3318 cm^−1^ verified O-H functionality. In the salicylidene-phenylhydrazine (**SPH**) Schiff base, a characteristic strong peak at 1614 cm^−1^ due to azomethine functionality (-C=N) confirmed its formation. In addition, a broad band of OH groups was also present at 3369–3345 cm^−1^, and the band at 1264 cm^−1^ indicated C-O functionality in the precursor SPH Schiff base.

In parahydroxy benzylideneaniline (**HBA**), the characteristic peak of the -C=N group was observed at 1621 cm^−1^, whereas the corresponding peaks of the C-O and C-N groups were observed at 1320 cm^−1^ and 1230 cm^−1^, respectively. A broad peak for the O-H group was observed between 3491 and 3317 cm^−1^.

### 3.3. FTIR Analysis via Supramolecular Interactions of 5-FU Cocrystals

Cocrystals are designated as self-assembly products formed because of an active component having pharmaceutical potential and a co-former [52]. A comparative study of the spectral details of the cocrystals and 5-fluorouracil was conducted. The outcomes of this study helped to reveal the engagement of core peaks’ N-H (hydrogen bond donor) and C=O (hydrogen bond acceptor) vibrations in the cocrystal 5-FU to hydrogen bonding. The characteristic peaks of -NH and -C=O of 5-FU all the synthesized cocrystals; 5-FU-BA, 5-FU-BU, 5-FU-SA, 5-FU-SPH and 5-FU-HBA are shown in Table 2. There appeared to be a blunt peak at 3220 cm^−1^ in the spectrum of 5-FU, which could be attributed to *v*(N-H), and a relatively high-intensity strong absorption band at 1662 cm^−1^, which could be assigned to -C=O modes. There is a significant impact of H-bonding on the chemical shifts of -N-H and -C=O vibrations of 5-FU, co-formers, and cocrystals [53].

### 3.4. 5-FU-BA

**Figure 1:**
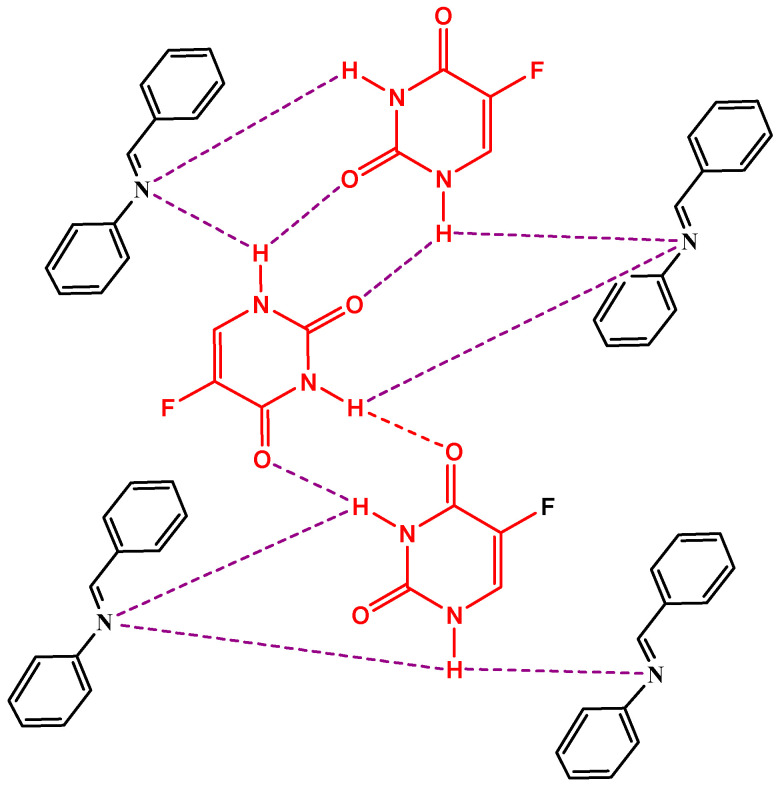
Proposed mode of interaction between 5-FU and BA Schiff base.

The shift of N-H vibrations in 5-FU moved to higher wavenumbers after cocrystallization, and the band shifted from 3220 cm^−1^ to 3248 cm^−1^ in the case of 5-FU-BA (Figure 1). This blueshift indicated the breakdown of intrinsic H-bonding connections in 5-FU and the formation of new ones. Interestingly, a similar high frequency was observed in the C=O stretching vibrations, where peaks moved from 1662 cm^−1^ to 1690 cm^−1^ owing to H-bonding, as shown in Figure S2. This leads to an interesting phenomenon that states that the cocrystal’s H-bonding and packing could be disrupted to create a higher energy solid with effective water solubility.

### 3.5. 5-FU-BU

**Figure 2:**
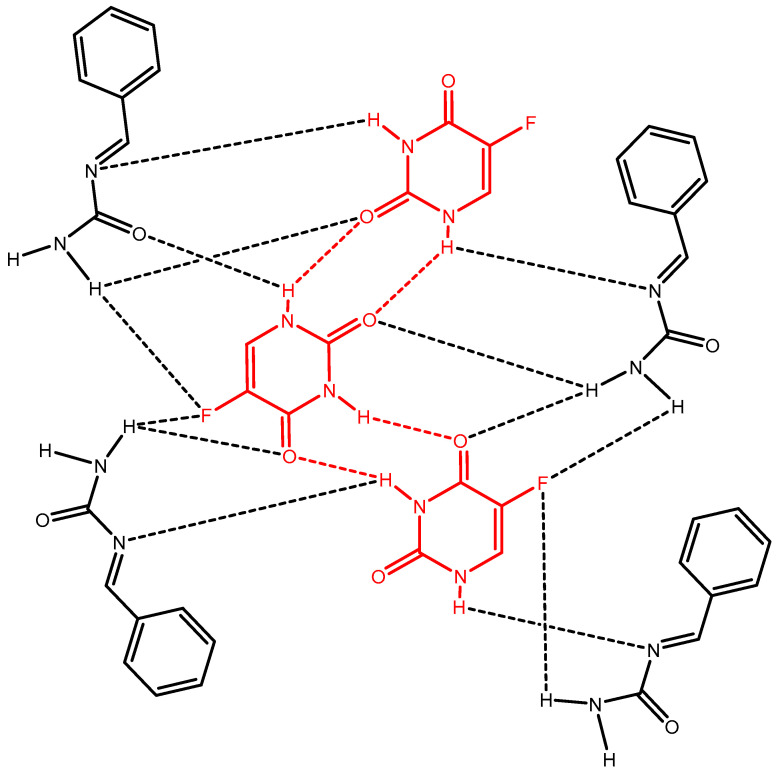
Proposed mode of interaction between 5-FU and the BU Schiff base.

In the 5-FU-BU cocrystal (Figure 2), there was also a blueshift in the absorption peak of the amino group to 3366 cm^−1^ and the C=O group to 1704 cm^−1^ as shown in Table 2. The imino (-C=N) group was shifted from 1613 cm^−1^ to 1670 as a result of H-bonding between 5-FU and benzylidene-urea.

### 3.6. 5-FU-SA

**Figure 3:**
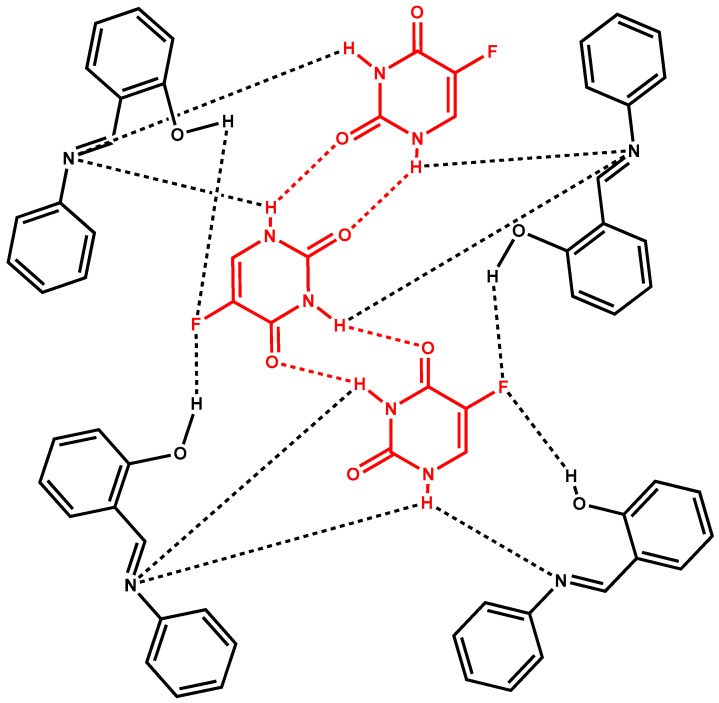
Proposed mode of interaction between 5-FU and the SA Schiff base.

As evinced from Figure 3, the absorption frequencies of the amino and carbonyl functionalities in 5FU-SA were observed at 3276 cm^−1^ and 1726 cm^−1^, respectively. H-bonding caused the shift of the C=N group to 1655.81 cm^−1^ as H-bonding significantly altered the vibrational modes of the functional groups.

### 3.7. 5-FU-SPH

**Figure 4:**
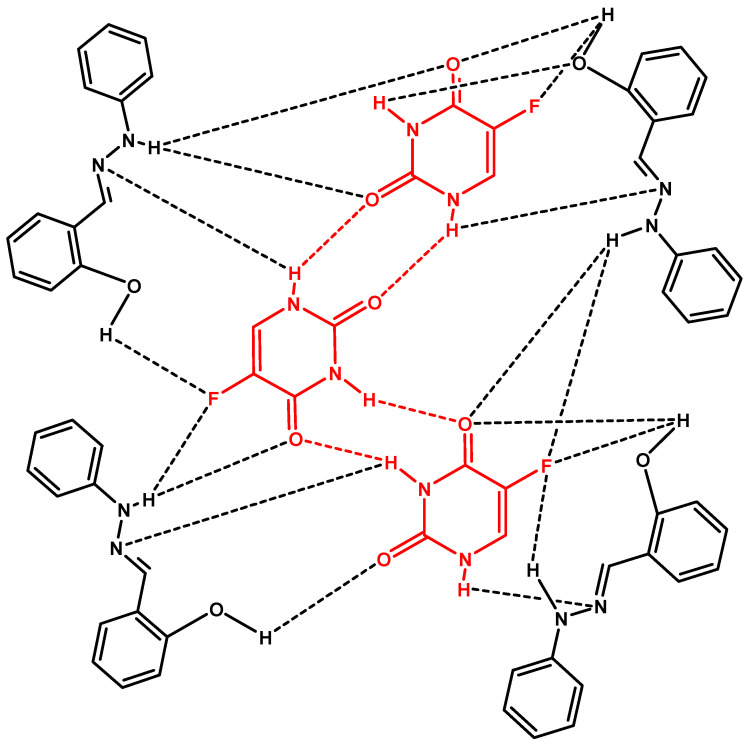
Proposed mode of interaction between 5-FU and the SPH Schiff base.

In 5FU-SPH (Figure 4), the absorption frequencies of amino and carbonyl functionalities were observed at 3304 cm^−1^ and 1746 cm^−1^, respectively. H-bonding caused the shift of the imino group in the Schiff base to 1628 cm^−1^.

### 3.8. 5-FU-HBA

**Figure 5:**
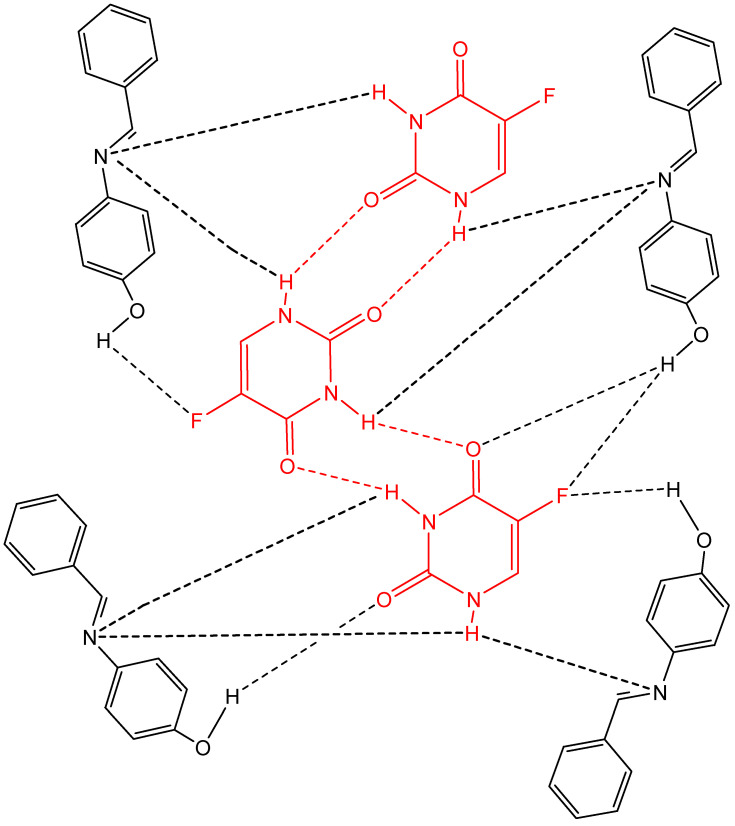
Proposed mode of interaction between 5-FU and the HBA Schiff base.

There was a shift of the amino and carbonyl peaks to 3311 cm^−1^ and 1753 cm^−1^ in this cocrystal compared with the corresponding peaks of 5-FU at 3220.24 cm^−1^ and 1662 cm^−1^, respectively (Figure 5). The peak of the imino group was also shifted to 1642 cm^−1^. All these shifts in the peaks of functional groups were caused by H-bonding between 5-FU and the co-former HBA. Thus, it can be concluded that substantial variations in the absorption frequencies of the predicted peaks in all cocrystals followed a similar pattern and led to the effective development of new noncovalent interactions.

### 3.9. Structural Analysis

**Figure 6:**
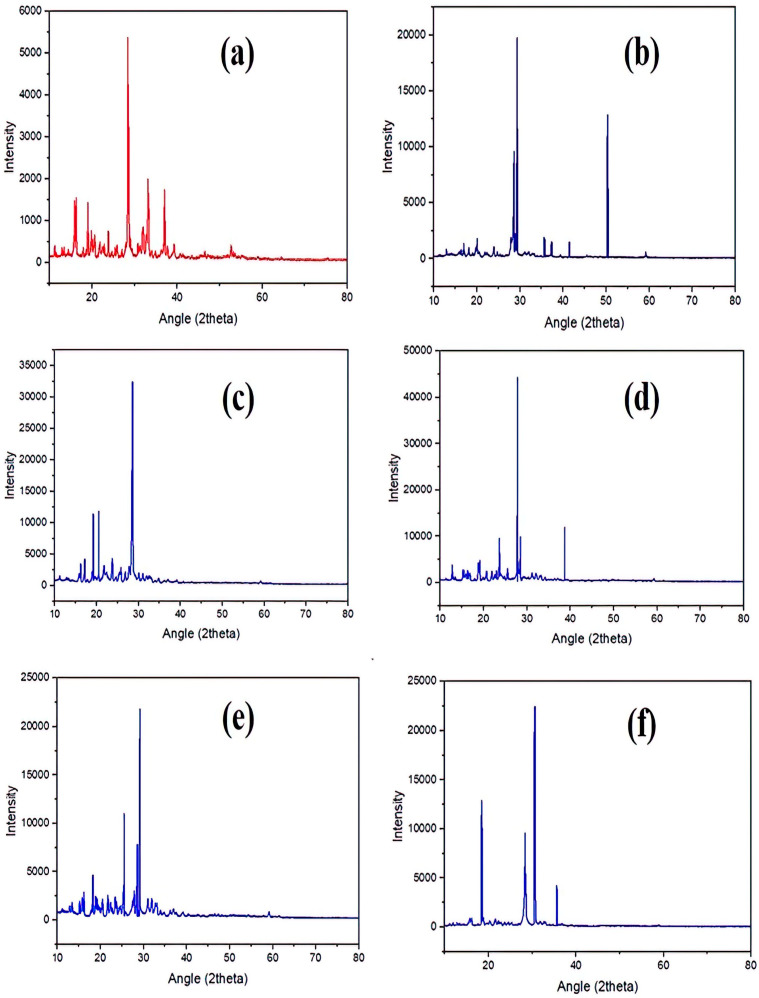
PXRD spectral analysis of (**a**) 5-FU; (**b**) 5-FU-BA; (**c**) 5-FU-BU; (**d**) 5-FU-SA; (**e**) 5-FU-SPH; and (**f**) 5-FU-HBA.

The structural characterization of cocrystals was further studied using powder XRD (Figure 6). This analysis revealed some significant shifting of 5-FU peaks in terms of shapes and intensities in all cocrystals. This peak shifting behavior of 5-FU was attributed to intermolecular interactions of precursor Schiff base co-formers. In addition, there were some new peaks observed in the 5-FU-cocrystal plots compared with typical 5-FU peaks [54]. These shifts were indicative of structural changes in 5-FU owing to changing molecular contacts with co-forming Schiff bases [35].

The most intense characteristic peak in 5-FU observed at 2θ = 28.36 agreed with the literature reported value, as seen in the stacked plot of 5-FU and its respective cocrystals with Schiff bases [39]. That peak showed an intensity of 5367, which showed a significant shift of peaks in terms of position and intensity.

The most significant and intense peak was observed at 2θ = 29.33 in the case of 5-FU-BA (Figure 6b) compared with the characteristic peak of 5-FU (Figure 6a). This manifestation showed the specific arrangement and preferred orientation of molecules in cocrystallization. In the second cocrystal 5-FU-BU (Figure 6c), a high-intensity (32,440) peak was observed at 2θ = 28.55 as a result of enhanced crystallinity compared with pure 5-FU. When cocrystal derivatives of SA (Figure 6d) and SPH (Figure 6e) with 5-FU were observed, the most intense respective peaks were seen at 2θ = 27.82 (intensity of 44,325) and 2θ =29.14 (intensity of 21,804), with comparatively higher intensities than that of pure 5-FU. All of the cocrystals showed higher intensity than API 5-FU, which indicated an enhanced specific orientation in crystal packing. Significant variation exists not only in the position, shapes, and intensity, but also in the FWHM (full width at half maximum), in comparison with 5-FU. While observing the 5-FU-HBA cocrystal (Figure 6f), its characteristic peak was present at 2θ = 30.59, with an intensity of 22,432. All comparative values of intensities, 2θ values, and FWHM values of all significant peaks are summarized in Table 3 for better comprehension and clarity.

### 3.10. In Vitro Anticancer Activity

The inhibition rate was used to study the trend of in vitro cell viability and pharmacological efficacy of 5-FU in its corresponding cocrystals against the colorectal carcinoma cell line SW-480 through an MTT assay3-(4,5-dimethyl-2-thiazolyl)-2,5-diphenyl-2*H*--tetrazolium bromide. Doxorubicin was the reference drug and the anticancer activity was compared with newly synthesized cocrystals of 5-FU and Schiff bases. Untreated cancer cell lines were the negative control, whereas the drug-solution-treated cell line was used as the positive control.

The resulting values showed that the concentration of standard drug and inhibition rate are directly dependent on each other; the higher the concentration, the higher the percent inhibition [45]. The rate of inhibition of SW480 cell proliferation by 5-FU cocrystals increased with the increasing drug dose. We investigated whether 5-FU-BA and 5-FU-HBA prominently induced apoptosis in SW480 cancer cells, and the rate of apoptosis increased as the concentration of these cocrystals increased.

At a concentration of 200 µg/mL, maximum growth inhibition was observed for 5-FU and its cocrystal forms. FU-BA showed remarkable inhibition even compared with the standard drug doxorubicin at lower concentrations. We synthesized this derivative, which has a great potential inhibition rate against SW480 anticancer cell lines. The IC_50_ values of 5-FU, 5-FU cocrystals, and the standard drug against cancerous cell lines are tabulated in Table 4. The results of the percentage inhibition rate at six doses against SW480 cell lines are summarized in Table 5.

The rate of inhibition response of the standard drug and 5-FU cocrystals was found to be concentration-dependent. The comparison of the inhibition rates and IC_50_ (µg/mL) with all of the cocrystals of 5-FU showed that 5-FU-BA and 5-FU-HBA have maximum anticancer potential at all concentrations against the SW480 colon cancer cell line. At a 200 µg/mL dose, 5-FU-BA showed anticancer potential with an IC_50_ value of 6.4731, which is significantly lower than that of 5-FU (IC50 = 12.1166), and almost comparable to the reference drug doxorubicin (IC50 = 2.3159) against SW480 cancer cell lines. Similarly, 5-FU-HBA exhibited an IC_50_ value of 10.2174. The inhibition rates of 5-FU-BA and 5-FU-HBA were highest among the derivatives (99.85% and 99.37%, respectively) in comparison with doxorubicin (97.103%). A significant increase in cell inhibition established the improvement in the efficacy of most prominent cocrystals in the following order: 5-FU-BA > 5-FU-HBA > 5-FU. These optimum results could be attributed to the readiness of API release on the target, and their improved efficacy is due to the pharmaceutical efficacy of the co-formers. There were varied interactions between the co-formers and 5-FU owing to their distinct structural features, which resulted in different inhibition rates.

In comparison with our formerly reported cocrystals of 5-FU with leucine, tryptophane, cinnamic acid, urea, thiourea, acetanilide, and aspirin, we prepared a new series of cocrystals of 5-FU to evaluate their efficacy toward the SW480 colon cancer cell line at varying concentrations in comparison with 5-FU [2,35,38,39].

### 3.11. Computational Studies

#### 3.11.1. TS (1HVY) Docked with 5-FU

The activities of the cocrystals were rationalized by a molecular modeling approach to envisage binding modes with the target cancer protein; moreover, all of the cocrystals showed interactions via H-bonding. The calculated interaction energies were then used to reveal the anticancer potential of the cocrystals.

**Figure 7:**
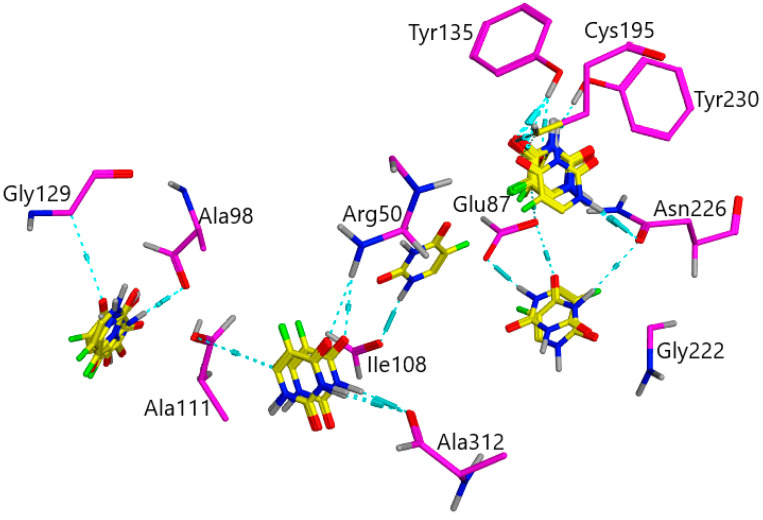
Interaction diagram of the protein–ligand complex of TS and 5-FU.

The docking results were imported into MOE to analyze the interaction between TS and 5-FU (Figure 7). However, the interaction analysis diagram showed that Arg50, Glu87, Ile108, Ala111, Tyr135, Asb226, and Ala312 are involved in the interaction of TS with 5-FU. However, based on the higher number of interactions and better GOLD fitness score, the best pose was selected in which Arg50 shares an H-bond with the carbonyl of 5-FU, while Ala111 and Ala312 also form an H-bond with the amino group of 5-fluorouracil. These interactions show that TS has the potential to efficiently bind with 5-FU and may act as a potent compound against colorectal cancer. 5-FU showed H-bonds with Arg50, Asn111, IIe108, Tyr135, Tyr230, Asn226, and Ala312, highlighted in yellow and pink, respectively.

#### 3.11.2. TS (1HVY) Docked with 5-Fluorouracil and Benzylidene-Urea (BU)

**Figure 8:**
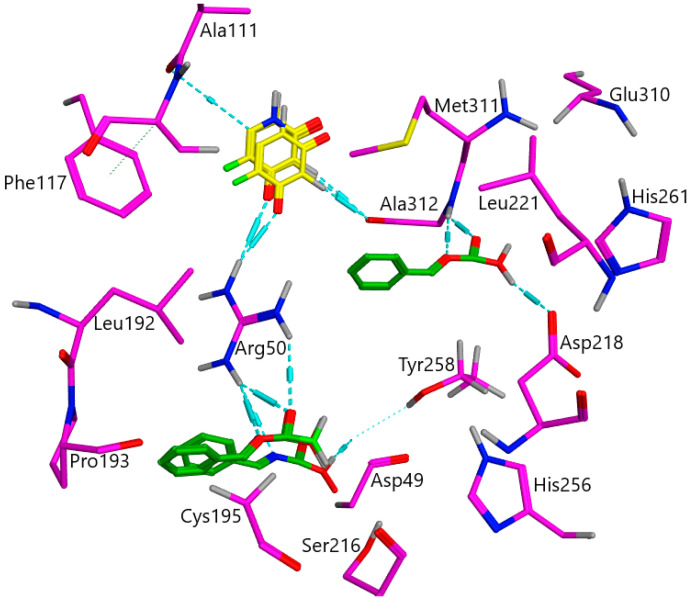
Interaction diagram of the protein–ligand complex of TS and 5-FU-BU.

The docking results in Figure 8 showed that Arg50 acts as a central atom between 5-FU and BU, forming an H-bond with the carbonyl group of 5-FU, while forming a strong H-bond with the carbonyl and R groups of BU, as shown in Figure 5. One of the docked poses of BU formed H-bond interactions with Ala312, which also acts as a central atom between 5-FU and BU. 5-FU forms H-bonds with Arg50, Asn112, and Ala312, while BU forms H-bonds with Arg50, Asp258, Asp218, and Ala312. TS, 10-FU, and BU are highlighted in pink, yellow, and green, respectively.

#### 3.11.3. TS (1HVY) Docked with 5-Fluorouracil and Salicylidene-Aniline (SA)

**Figure 9:**
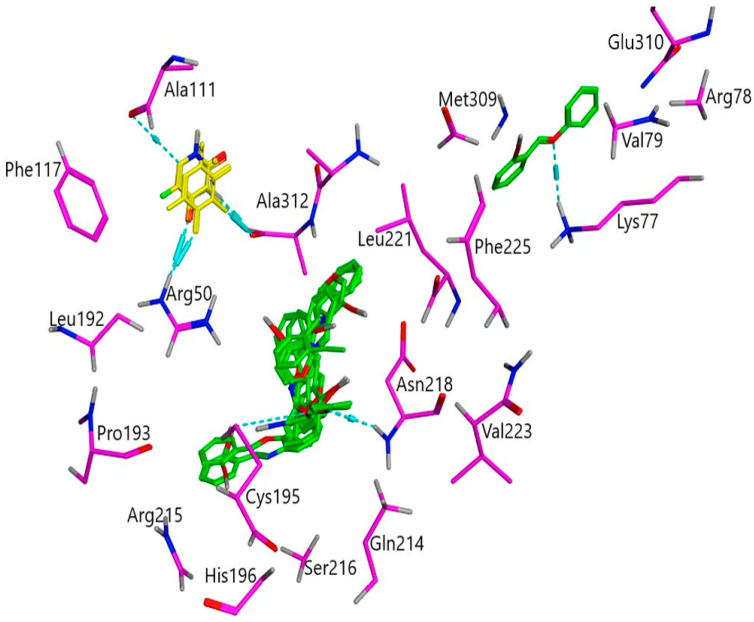
Interaction diagram of the protein–ligand complex of TS and 5-FU-SA.

The interaction analysis diagram (Figure 9) shows that most of the docked poses of salicylideneaniline formed H-bonds with Cys195 and Asn218, while one of the poses of salicylideneaniline showed H-bond interactions with Lys77. 11-FU shows H-bonds with Arg50, Asn112, and Ala312, while SA shows interactions with Lys77, Cys195, and Asn218. TS, 5-FU, and SA are highlighted in pink, yellow, and green, respectively.

#### 3.11.4. TS (1HVY) Docked with 5-Fluorouracil and Salicylidene-Phenylhydrazine (SPH)

**Figure 10:**
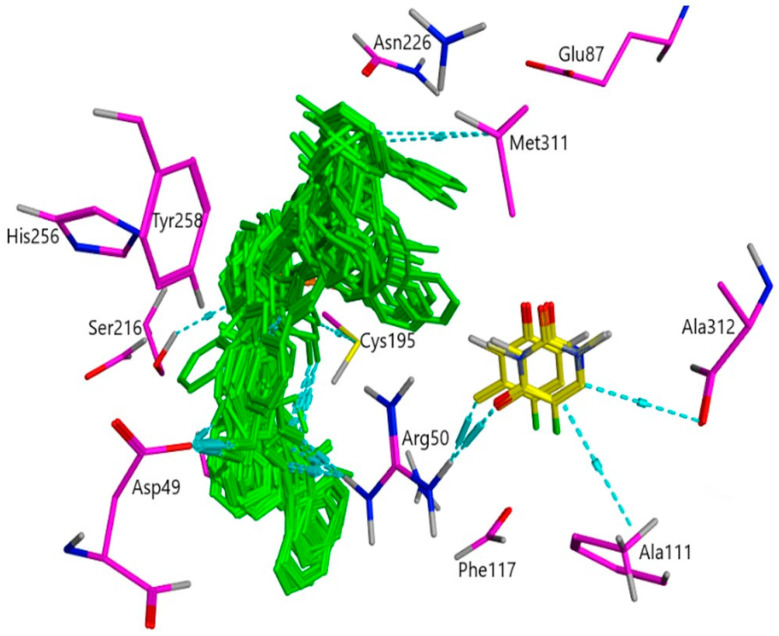
Interaction diagram of the protein–ligand complex of TS and 5-FU-SPH.

All of the docked poses were generated within the binding cavity of TS, forming a cluster, as shown in Figure 10. The interaction analysis diagram of 5-FU and SPH showed that TS strongly binds with the complex and forms a greater number of interactions, which elucidates that the 5-FU-SPH complex may act as a potent compound against colorectal cancer cell lines, which also strengthens our in vitro results. The diagram shows that 5′-fluorouracil and salicylidenephenyl-hydrazine share H-bonds with Asp49, Arg50, Ala111, Ala312, Met311, Cys195, and Ser216.

#### 3.11.5. TS (1HVY) Docked with 5-Fluorouracil and Para-hydroxy Benzylideneaniline (HBA)

**Figure 11:**
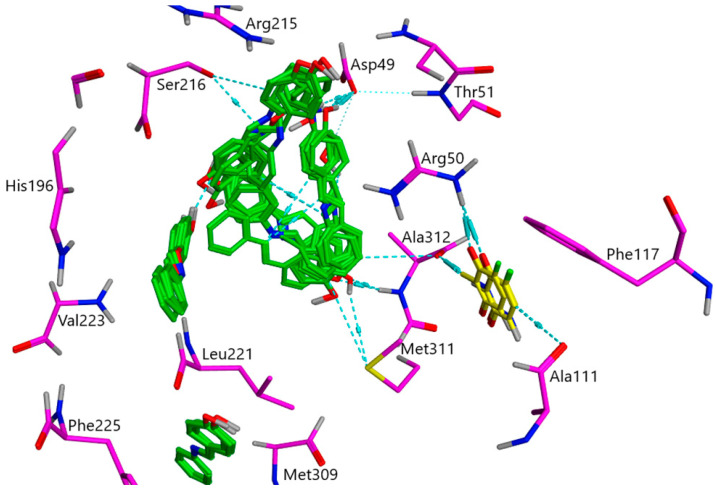
Interaction diagram of the protein–ligand complex of TS and 5-FU-HBA.

The docking results in Figure 11 showed that the 5-FU-HBA complex binds with TS, forming an H-bond with various binding site residues, while some of the docked poses were generated away from the binding cavity of TS.

## 4. Conclusions

Five new cocrystals of 5-FU were prepared by the thermal condensation method using Schiff bases: benzylidene-urea (**BU**), benzylidene-aniline (**BA**), salicylidene-aniline (**SA**), salicylidene-phenylhydrazine (**SPH**), and para-hydroxy benzylideneaniline (**HBA**). Once their pharmacokinetic effects and subsequent metabolites were properly examined, the four co-formers were chosen. Five different Schiff bases were prepared by condensation reaction using glacial acetic acid as a catalyst, and this confirmation was performed by FTIR analysis. Then, successful cocrystals were produced using the grinding method with a few drops of distilled water, and there was no requirement for extreme temperature conditions. The FTIR and PXRD data confirmed this. No side product is formed in cocrystal formation. In comparing the spectra of the active pharmaceutical ingredient (API), i.e., 5-FU and cocrystals, it was possible to see considerable changes in the predicted 5-FU peak positions using characterization approaches. The primary peaks of interest, amino (3146 cm^−1^) and carbonyl (1671 cm^−1^), were considerably displaced in all of the cocrystals. FTIR spectra were compared to the spectrum of 5-FU, in line with the pattern noted in the literature. Cocrystal production was successfully verified by PXRD spectra as well. Anticancer assays of the synthesized prodrugs were also carried out against HCCT 116 cell lines. In the MTT assay, all of the cocrystals demonstrated anti-HCCT 116 cell line activity, and the results were reinforced by molecular modeling studies indicating stable binding energies of the synthesized cocrystals with the protein. In short, it can be assumed that cocrystallization has several benefits over other processes, including ease of manufacture, lack of byproduct synthesis, and clarity. This method can be used for further discoveries in cancer treatment.

## Figures and Tables

**Table 1:** FTIR and physical appearance observation of precursor Schiff bases.

Sr. No.	Schiff Base	C=N Stretch (cm^−1^)	Physical Appearance of Schiff Base Crystals
1	Benzylideneaniline (**BA**)	1607	White
2	Benzylideneurea (**BU**)	1613	Yellowish white
3	Salicylideneaniline (**SA**)	1593	Yellow
4	Salicylidenephenyl-hydrazine (**SPH**)	1614	Yellow
5	Parahydroxy Benzylidene-aniline (**HBA**)	1621	White

**Table 2:** Absorption peaks of 5-FU and its cocrystals with Schiff bases.

Sr. No.	Sample ID	V(N-H) cm^−1^	V(C=O) cm^−1^
1	5-FU	3220	1662
2	5-FU-BA	3248	1690
3	5-FU-BU	3366	1704
4	5-FU-SA	3324	1795
5	5-FU-SPH	3304	1746
6	5-FU-HBA	3311	1753

**Table 3:** PXRD analysis of 5-FU and its corresponding cocrystals with Schiff bases.

Sr. No.	Sample	2 θ	Intensity	FWHM	Crystalline Size (nm)
1	5-FU	28.37	5367	0.28	30.58
2	5-FU-BA	29.34	19,748	0.15	57.20
3	5-FU-BU	28.56	32,440	0.25	34.26
4	5-FU-SA	27.82	44,325	0.09	95.02
5	5-FU-SPH	29.15	21,804	0.12	71.47
6	5-FU-HBA	30.59	22,432	0.21	40.98

**Table 4:** IC_50_ values of 5-FU and its cocrystals compared with the standard drug.

IC_50_ (µg/mL) against Cell Lines		
Sample	Compounds	SW-480
1	5-FU	12.1166
2	5-FU-BA *	**6.4731**
3	5-FU-BU	13.5591
4	5-FU-SA	97.1558
5	5-FU-SPH	11.6904
6	5-FU-HBA *	**10.2174**
7	Standard Drug (Doxorubicin)	2.3159

* Compounds **2** and **6** are potent enough to be used for further evaluation for the treatment of colorectal cancer.

**Table 5:** Inhibition rate (%) of synthesized cocrystals of 5-FU and Schiff bases against the colorectal carcinoma cell line SW480.

Inhibition Rate (%) against SW480 Cell Line							
Conc. (µg/mL)	1	2	3	4	5	6	Doxorubicin
200	97.4047	99.8492	88.5714	60.7540	95.6905	99.3650	97.1032
100	94.0476	95.6587	87.5000	57.1587	95.1111	95.7936	93.8889
50	80.1984	93.2540	87.8968	46.0079	94.7064	82.14286	92.2222
25	70.4365	81.7857	87.6191	39.4762	92.5555	65.5555	90.9524
12.5	52.5794	76.2302	13.0476	30.3968	77.0238	52.7381	84.9603
6.25	7.3413	28.3333	25.9524	17.6587	37.8730	21.58730	38.7698

Compounds **2** and **6** have shown good inhibition when compared with the standard drug doxorubicin.

## Data Availability

All the raw data of this research can be obtained from the corresponding authors upon reasonable request.

## Supplementary Materials

The following supporting information can be downloaded at https://www.mdpi.com/article/10.3390/pharmaceutics15071929/s1. Figure S1: FTIR analysis of Schiff bases (a); BA (b); BU (c); SA (d); SPH (e) HBA; Figure S2: Comparative FTIR analysis of 5-FU and Schiff bases with corresponding cocrystals.
