# Supplementary material for: Synthesis, Characterization, Theoretical and Experimental Anticancer Evaluation of Novel Cocrystals of 5-Fluorouracil and Schiff Bases against SW480 Colorectal Carcinoma
Source: Pharmaceutics. 2023 Jul 11;15(7):1929. doi: 10.3390/pharmaceutics15071929 (PMC10383612; doi:10.3390/pharmaceutics15071929)
Supplement: Supplementary file 1 [file pharmaceutics-15-01929-s001.zip › pharmaceutics-2442282-supplementary.pdf]

Synthesis, Characterization, Theoretical and Experimental Anticancer Evaluation of Novel Cocrystals of 5-Fluorouracil and Schiff bases against SW480 Colorectal Carcinoma

Farhat Jubeen[1], Ishrat Jubeen[2], Usman Aftab[3], Sadia Noor[2,4], Mah e Hareem[2], Misbah Sultan[5*], Mohsin Kazi[6*]

Supplementary Material

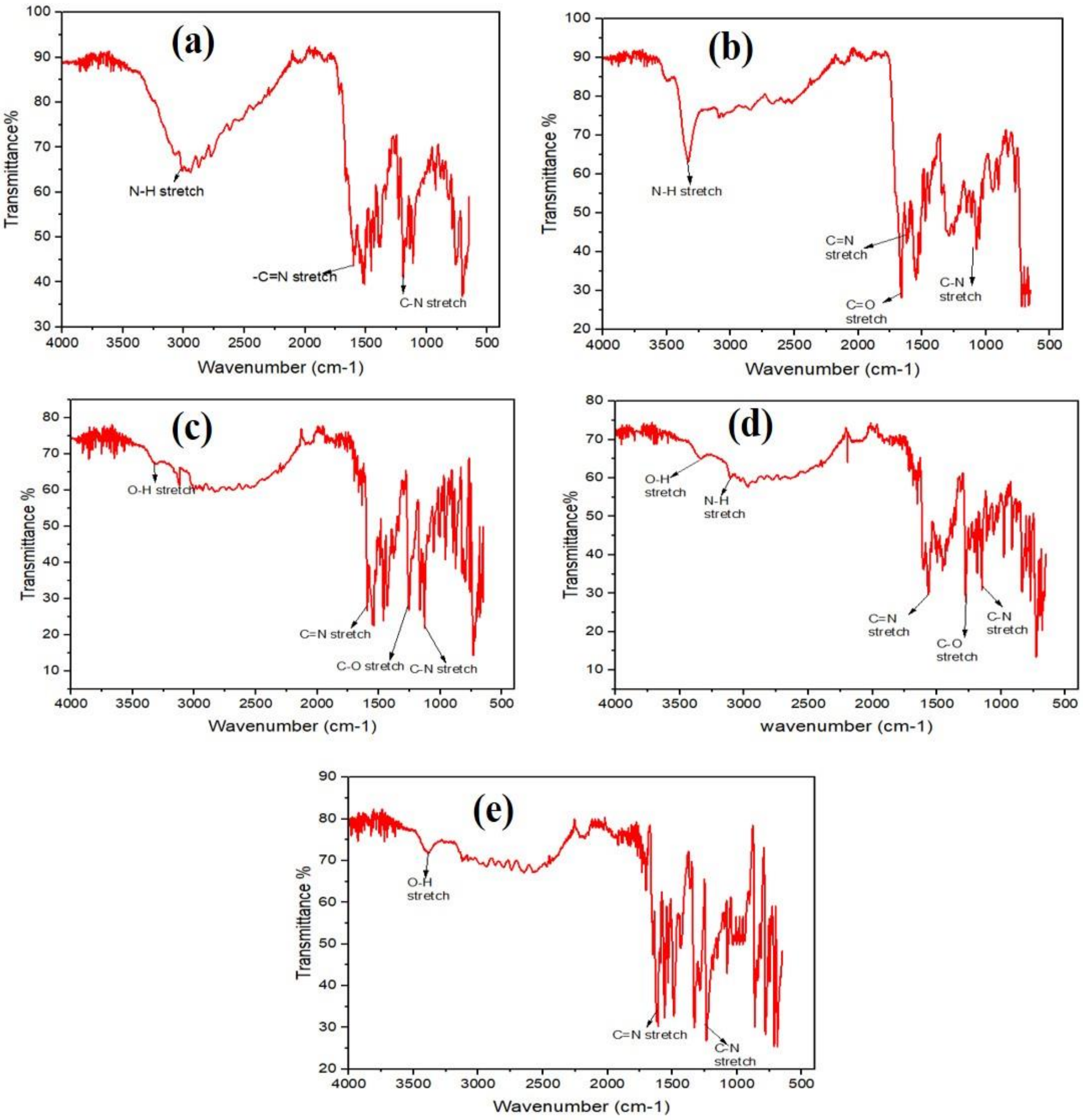

Figure S1: FTIR analysis of Schiff bases (a); BA (b); BU (c); SA (d); SPH (e) HBA

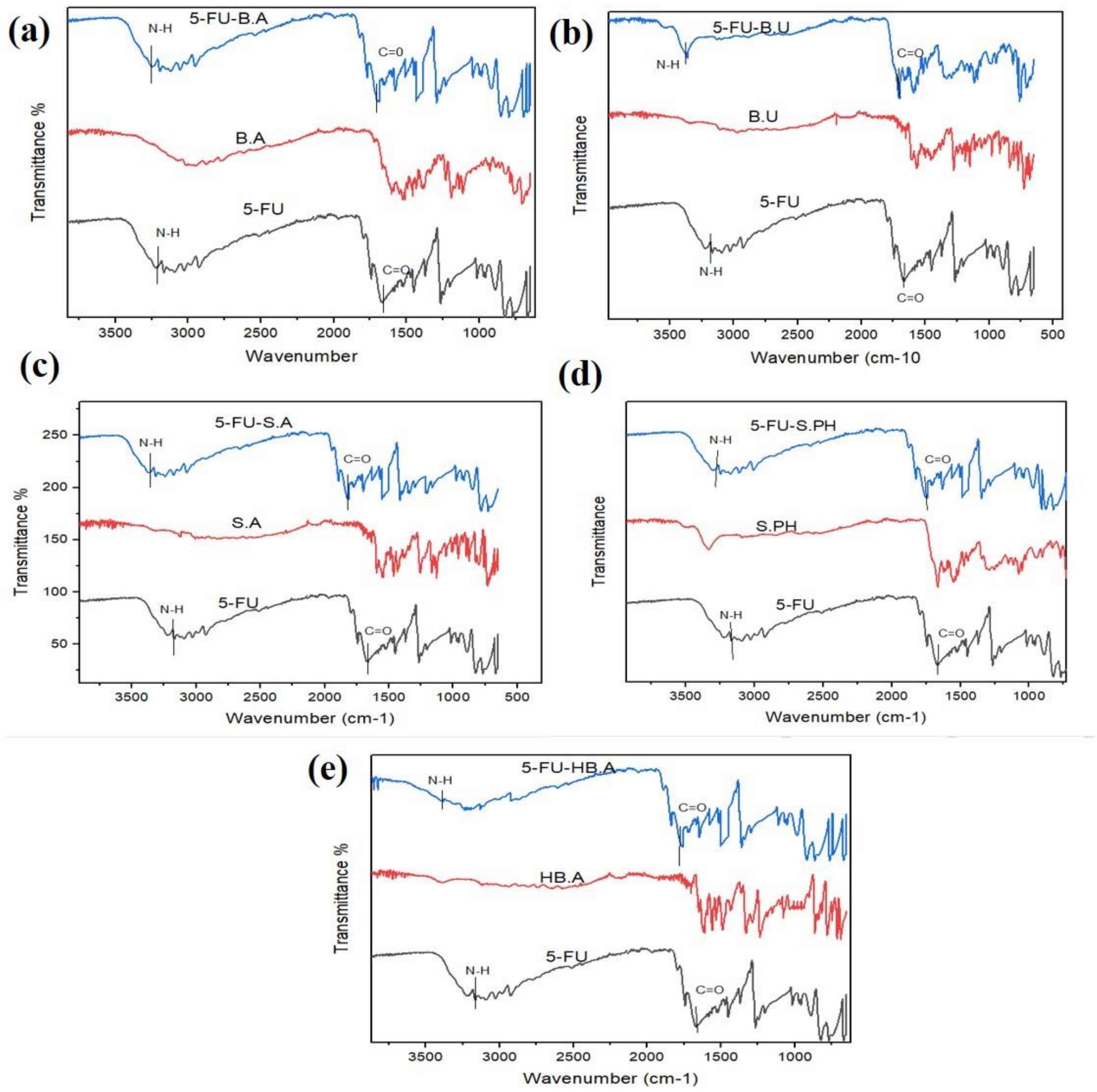

Figure S2: Comparative FTIR analysis of 5-FU and Schiff bases with corresponding cocrystals: (a) 5FU-BA; (b) 5FU-BU; (c) 5FU-SA; (d) 5FU-SPH; (e) 5FU-HBA
